# Supplementary material for: Social bonds decrease epigenetic age in male bottlenose dolphins
Source: Commun Biol. 2025 Nov 29;8:1765. doi: 10.1038/s42003-025-09227-w (PMC12700864; doi:10.1038/s42003-025-09227-w)
Supplement: Supplementary file 2 — Supplementary Information [file 42003_2025_9227_MOESM2_ESM.pdf]

Supplementary Information

Supplementary Note

Robustness of Results Across Various Epigenetic Clocks

Our findings could not be replicated when other epigenetic clocks were applied to our data. However, we noticed that in two clocks (Barratclough skin and Robeck general) that were calibrated on common bottlenose dolphins (*T. truncatus*), normalised node strength was approaching significance, thereby producing the most similar results compared to those generated using our LOIOCV approach using Indo-Pacific bottlenose dolphin (*T.aduncus*) samples. We hypothesise that clocks calibrated on a distantly related taxon or multi-species clocks may be able to infer which samples belong to older and younger animals but accurate chronological, and thus biological age estimates require accurate, species-specific clocks.

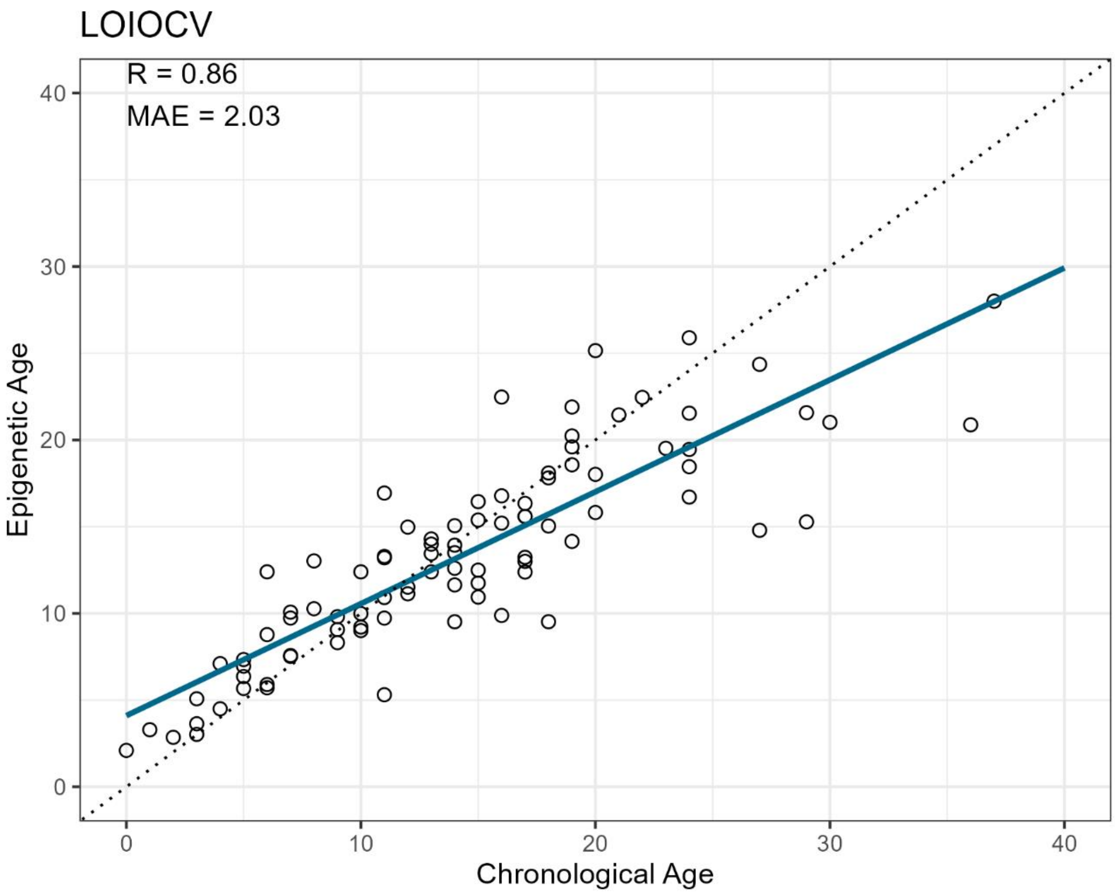

Supplementary Fig. 1. Epigenetic age versus chronological age for Shark Bay Indo-Pacific bottlenose dolphins using our LOIOCV approach. Epigenetic ages calculated for 68 individuals totalling 90 samples using elastic net regression models with leave-one-individual-out cross validation (LOIOCV), including precision (R) and accuracy in years (MAE) for this epigenetic clock. The regression line is shown in blue. The dotted diagonal line indicates epigenetic age = chronological age.

Model Selection

Supplementary Table 1. Random effects

Model	AIC
EpiAge ~ 1	307.36
EpiAge ~ 1 + (1|DolphinID)	307.42
EpiAge ~ 1 + (1|YearSampled)	300.68
EpiAge ~ 1 + (1|DolphinID) + (1|YearSampled)	295.47*

* Model with lowest AIC

Supplementary Table 2. Fixed effects

Model	AIC
EpiAge ~ 1 + (1|DolphinID) + (1|YearSampled)	298.11
EpiAge ~ Age + (1|DolphinID) + (1|YearSampled)	254.10
EpiAge ~ Age + NodeStrength + (1|DolphinID) + (1|YearSampled)	254.55
**EpiAge ~ Age + NodeStrength + GroupSize + (1|DolphinID) + (1|YearSampled)**	250.76*
EpiAge ~ Age + NodeStrength + GroupSize + CV + (1|DolphinID) + (1|YearSampled)	252.57
EpiAge ~ Age + NodeStrength*GroupSize + CV + (1|DolphinID) + (1|YearSampled)	254.26
EpiAge ~ Age + GroupSize + (1|DolphinID) + (1|YearSampled)	254.20
EpiAge ~ Age + NodeStrength*GroupSize + (1|DolphinID) + (1|YearSampled)	252.41

* Model with lowest AIC

Supplementary Table 3. Collinearity (Variance inflation factors, VIFs) and correlation coefficients between the variables included in the most supported model. VIFs for each variable are in the first row in italics, correlation coefficients below the diagonal.

	**Node Strength**	**Male Group Size**	**Chronological Age**
**Node Strength**	*2.951*	*1.623*	*2.357*
**Male Group Size**	0.618		
**Chronological Age**	0.742	0.429	

Mediation Analysis

Methods

To investigate the possibility of a more complex causal relationship between key predictors and outcome variables in our main analysis, we conducted a mediation analysis to test whether social bond strength mediates the relationship between male group size and epigenetic age, while controlling for chronological age (AgeDB). The analysis was performed using the ‘mediation’ package in R. We fitted three linear models:

1. Total effect model: Estimating the effect of group size on epigenetic age
2. Mediator model: Estimating the effect of group size on bond strength
3. Full model: Estimating the effects of both group size and bond strength on epigenetic age

Mediation was assessed using non-parametric bootstrapping with 1000 simulations to estimate confidence intervals. For the sake of computability, the mediation analysis was carried out on a simple regression model (no random effects) and not a linear mixed model.

Results

Supplementary Table 4. Mediation analysis – key results.

**Effect**	**Estimate**	**95% CI Lower**	**95% CI Upper**	***p*-value**
ACME (Indirect)	-0.422	-1.096	0.00	0.052
ADE (Direct)	0.696	0.052	1.49	0.036*
Total Effect	0.275	-0.398	0.90	0.476

*Significant at $p < 0.05$

The average causal mediation effect (ACME) was not significant ($p = 0.052$), suggesting a lack of strong evidence for social bond strength mediating the effect between group size and epigenetic age. The average direct effect (ADE), however, was significant ($p = 0.036$), indicating that group size had a direct effect on epigenetic age independent of bond strength.

Given the lack of compelling evidence for mediation in this simplified framework, we did not pursue more complex mediation models (e.g., structural equation models or mixed-effects mediation models) for the main analyses. Instead, we focused on the direct effects of both variables as reported in the main text.

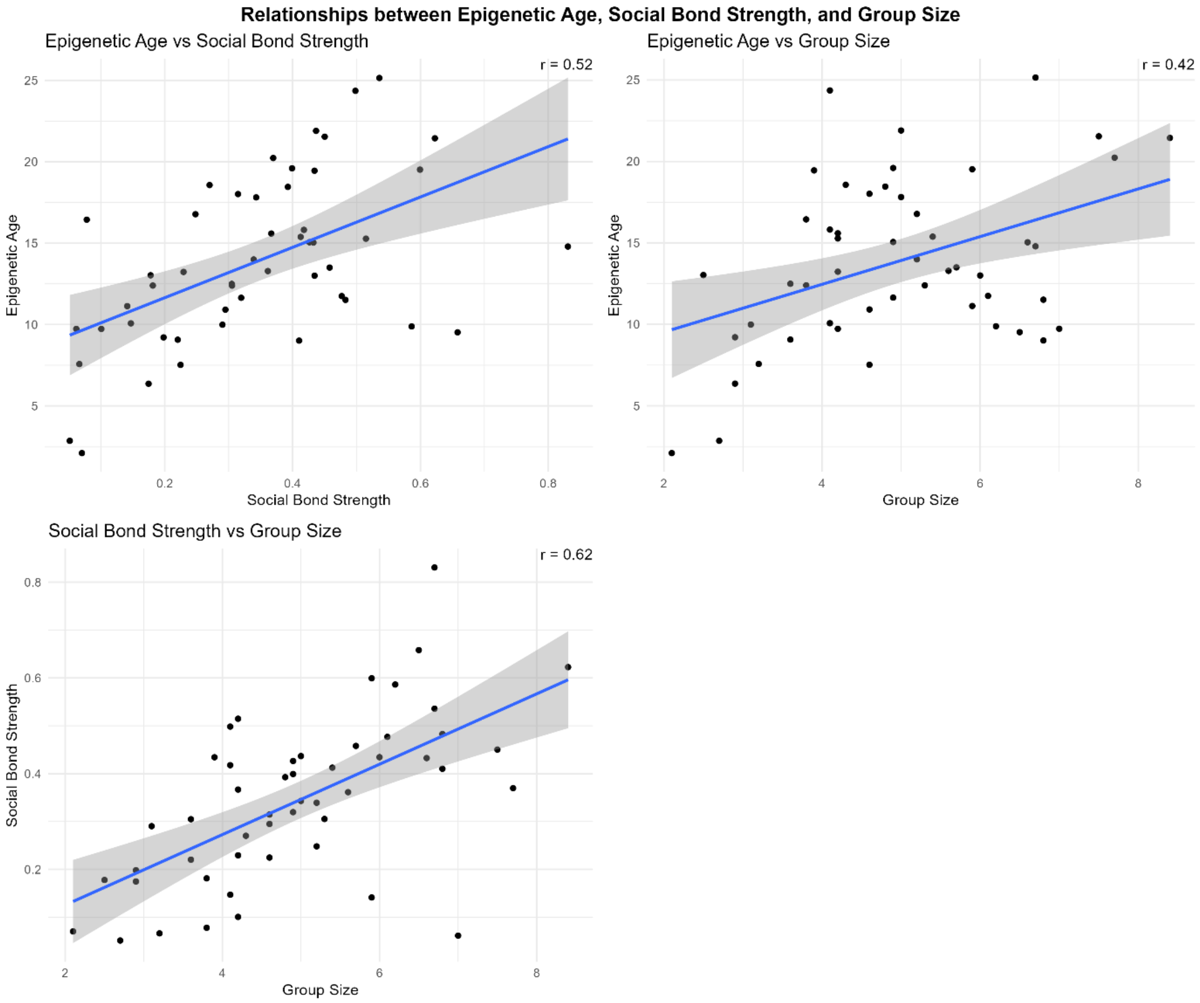

Supplementary Fig. 2. Pairwise scatterplots comparing epigenetic age, social bond strength, and group size.

From the raw data, a positive relationship is apparent between epigenetic age and social bond strength, though this relationship is negative in the best fitting global model. This apparent contradiction is driven by the confounding effect of chronological age [1], which is positively associated with both bond strength and epigenetic age. When this confounding effect is controlled for in the broader linear mixed model, the unique association between social bond strength and epigenetic age is revealed to be negative, when accounting for the other random and fixed effects in the model.

Supplementary Table 5. Results of LMM restricted to males whose chronological age was known with an accuracy of ± 6 months.

**Predictor variable**	**b**	***p*-value**
Epigenetic age<br>*($N_{Samples}$= 37, N_{Ind}= 27)*		
Intercept	2.46	0.28
Cumulative social bond strength	-11.25	0.037*
Mean male group size	0.75	0.14
Chronological age	0.80	<0.0001*

*Significant at $p < 0.05$

Supplementary Table 6. Results of LMM restricted to adult males ≥ 14 years old.

**Predictor variable**	**b**	***p*-value**
Epigenetic age *($N_{Samples}$= 30, N_{Ind}= 24)*		
Intercept	4.72	0.25
Cumulative social bond strength	-12.66	0.03*
Mean male group size	0.78	0.19
Chronological age	0.68	<0.0001*

*Significant at $p < 0.05$

Supplementary Table 7. Results of the LMM using age acceleration (AgeAccel) as response variable.

**Predictor variable**	**b**	***p*-value**
AgeAccel<br><br>*($N_{Samples}$= 50, N_{Ind}= 38)*		
Intercept	-2.41	0.19
Cumulative social bond strength	-9.84	0.015*
Mean male group size	0.92	0.015*
Chronological age	0.96	0.33

*Significant at $p < 0.05$

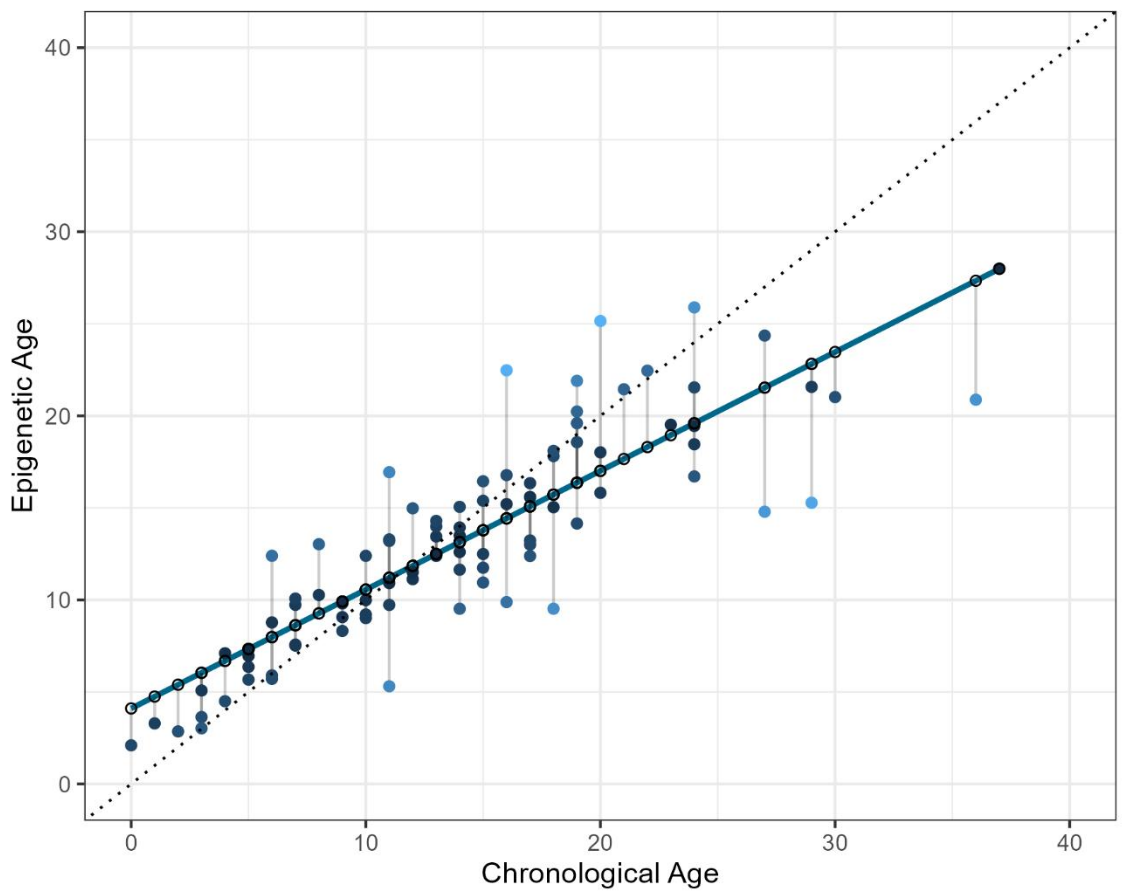

Supplementary Fig. 3. Epigenetic age versus chronological age for Shark Bay Indo-Pacific bottlenose dolphins (*Tursiops aduncus*) indicating age acceleration (AgeAccel) per individual. Epigenetic ages are calculated for 68 individuals totalling 90 samples, analysed using our LOIOCV approach (Supplementary Fig. 1). Age acceleration (AgeAccel) is indicated for each individual, representing the residuals of the regression of epigenetic age on chronological age (regression line in blue). Samples below the regression line indicate individuals with negative residuals (slower ageing), while the positive residuals suggest accelerated ageing. The samples closest to the regression line are dark blue, samples further away are coloured in lighter shades of blue. The black dotted diagonal line indicates epigenetic age = chronological age.

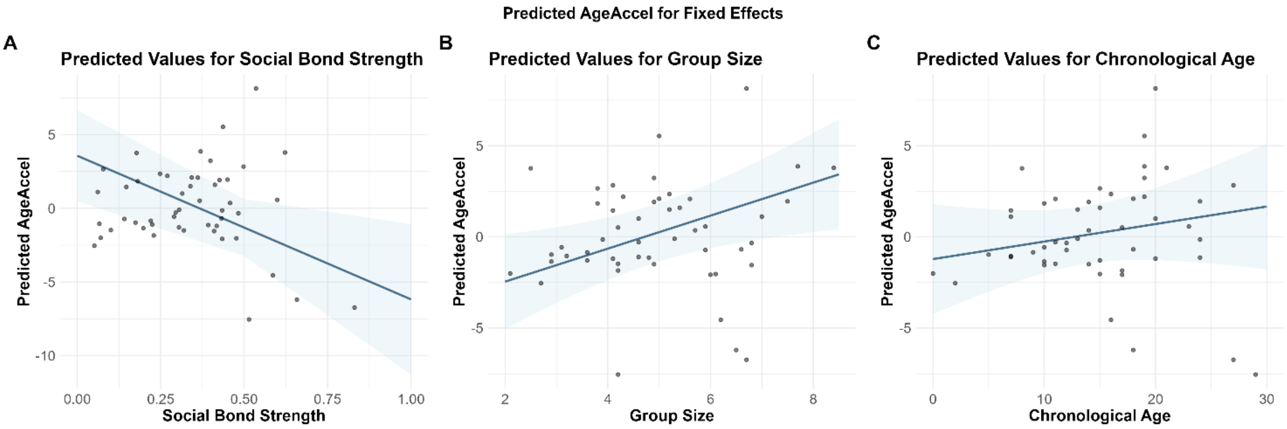

Supplementary Fig. 4. Predicted AgeAccel as a function of chronological age, social bond strength, and group size in Indo-Pacific bottlenose dolphins (*Tursiops aduncus*). Raw data (black dots) and model predictions across the dataset predicting AgeAccel based on the three fixed effects (chronological age, cumulative social bond strength, and group size). Each panel represents the relationship between one predictor and the predicted AgeAccel value, while controlling for other variables in the model. (A) Significant negative correlation between social bond strength and predicted AgeAccel. (B) Significant positive relationship between group size versus predicted AgeAccel. (C) Non-significant positive correlation between chronological age and AgeAccel. Blue lines represent the model's predicted values, with light blue ribbons indicating 95% confidence intervals. Random effects for individual dolphin ID and year sampled were included in the model but are not visualized here.

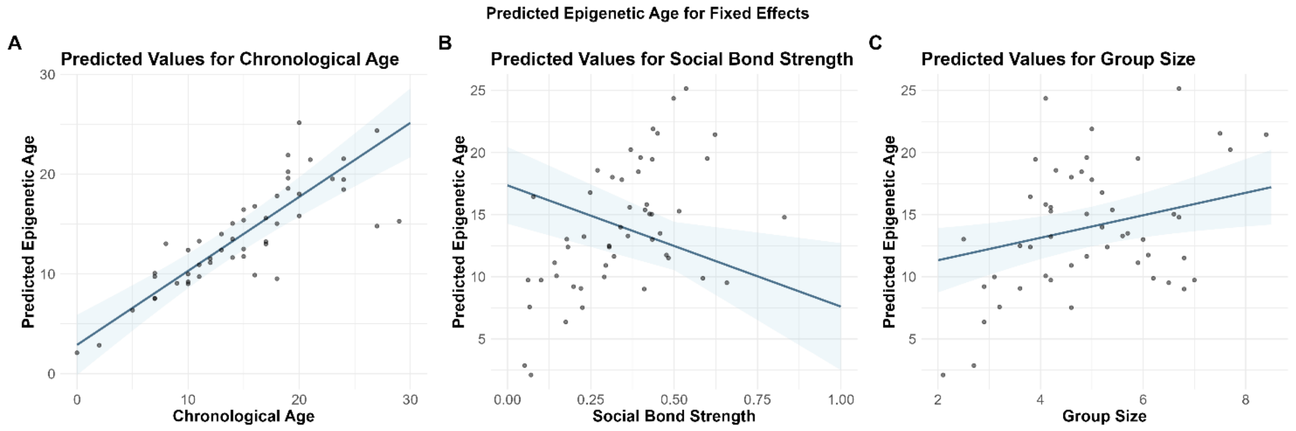

Supplementary Fig. 5. Predicted relationships between epigenetic age and chronological age, social bond strength, and group size in Indo-Pacific bottlenose dolphins (*Tursiops aduncus*). Raw data (black dots) and model predictions across the dataset predicting epigenetic age based on the three fixed effects (chronological age, cumulative social bond strength, and group size). Each panel represents the relationship between one predictor and the predicted epigenetic age, while controlling for other variables in the model. (A) Strong positive relationship of chronological age versus predicted epigenetic age. (B) Negative correlation between social bond strength and predicted epigenetic age. (C) Positive relationship between group size versus epigenetic age. Blue lines represent the model's predictive values, with light blue ribbons indicating 95% confidence intervals. Random effects for individual dolphin ID and year sampled were included in the model but are not visualised here.

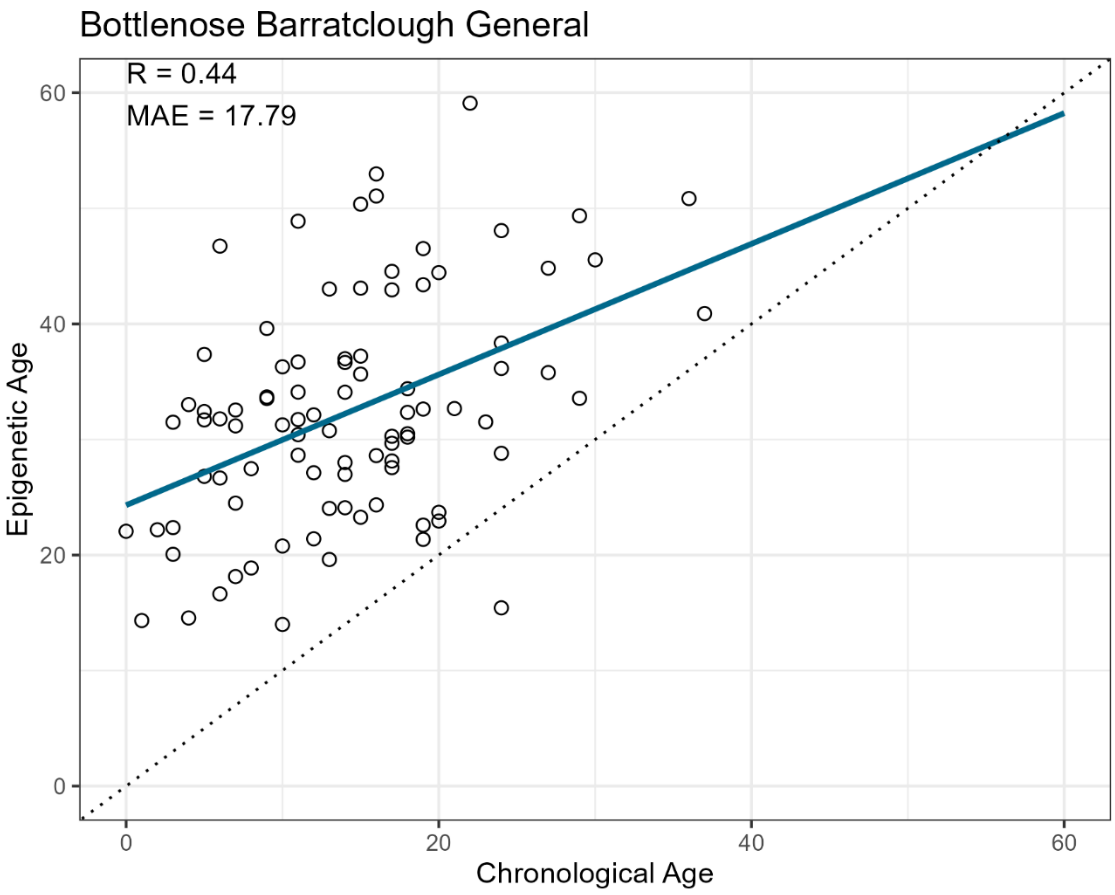

Supplementary Fig. 6. Epigenetic age versus chronological age for Shark Bay Indo-Pacific bottlenose dolphins (*Tursiops aduncus*) using Barratclough et al.'s (2021) bottlenose dolphin general clock. Epigenetic ages calculated for 68 individuals totaling 90 samples which were included in our calibration dataset, including precision (R) and accuracy in years (MAE) for this epigenetic clock. The regression line is shown in blue. The dotted diagonal line indicates epigenetic age = chronological age.

Supplementary Table 8. Results of LMM using epigenetic age estimates from Barratclough et al.'s (2021) bottlenose dolphin general clock [2].

**Predictor variable**	**b**	***p*-value**
Epigenetic age *($N_{Samples}$= 50, N_{Ind}= 38)*		
Intercept	25.07	<0.001
Cumulative social bond strength	-6.60	0.60
Mean male group size	-0.30	0.78
Chronological age		0.02*

*Significant at $p < 0.05$

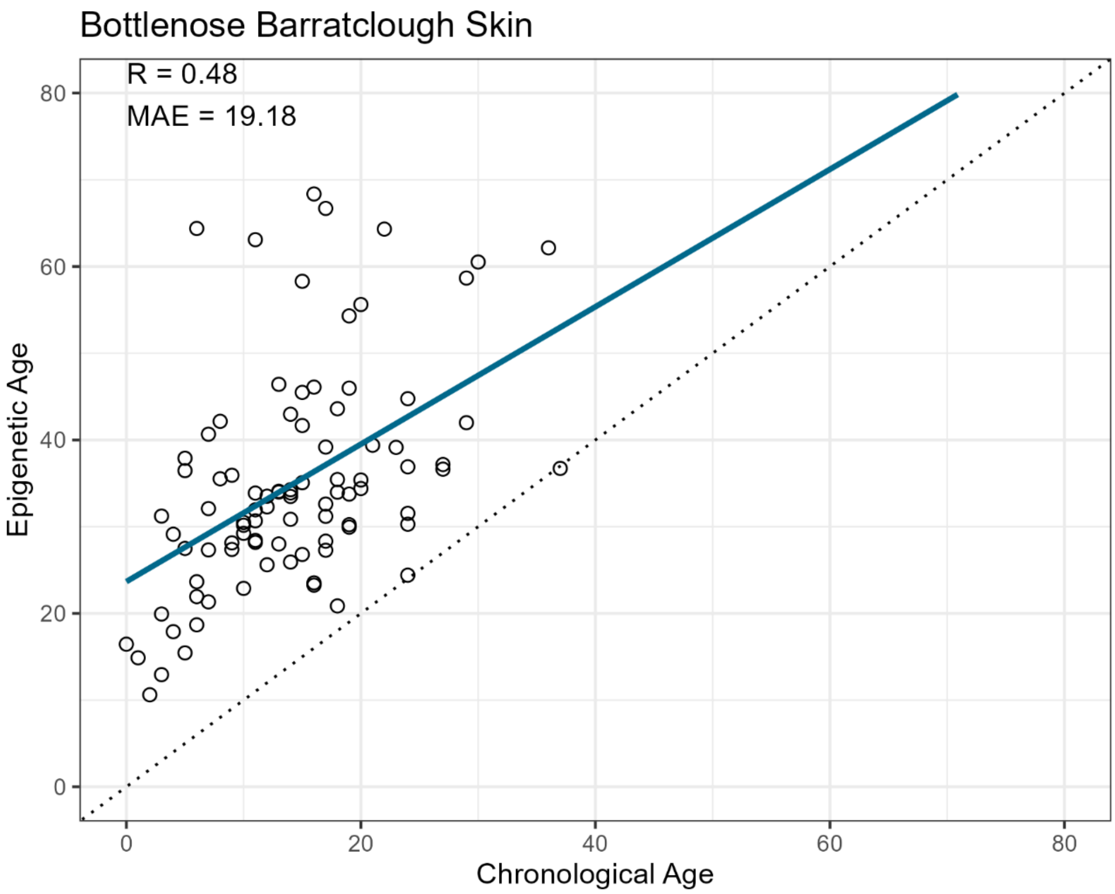

Supplementary Fig. 7. Epigenetic age versus chronological age for Shark Bay Indo-Pacific bottlenose dolphins (*Tursiops aduncus*) using Barratclough et al.'s (2021) bottlenose dolphin skin clock. Epigenetic ages calculated for 68 individuals totaling 90 samples which were included in our calibration dataset, including precision (R) and accuracy in years (MAE) for this epigenetic clock. The regression line is shown in blue. The dotted diagonal line indicates epigenetic age = chronological age.

Supplementary Table 9. Results of LMM using epigenetic age estimates from Barratclough et al.'s (2021) bottlenose dolphin skin clock [2].

**Predictor variable**	**b**	***p*-value**
Epigenetic age *($N_{Samples}$= 50, N_{Ind}= 38)*		
Intercept	21.44	<0.001*
Cumulative social bond strength	-25.19	0.07
Mean male group size	0.90	0.45
Chronological age	1.17	<0.001*

*Significant at $p < 0.05$

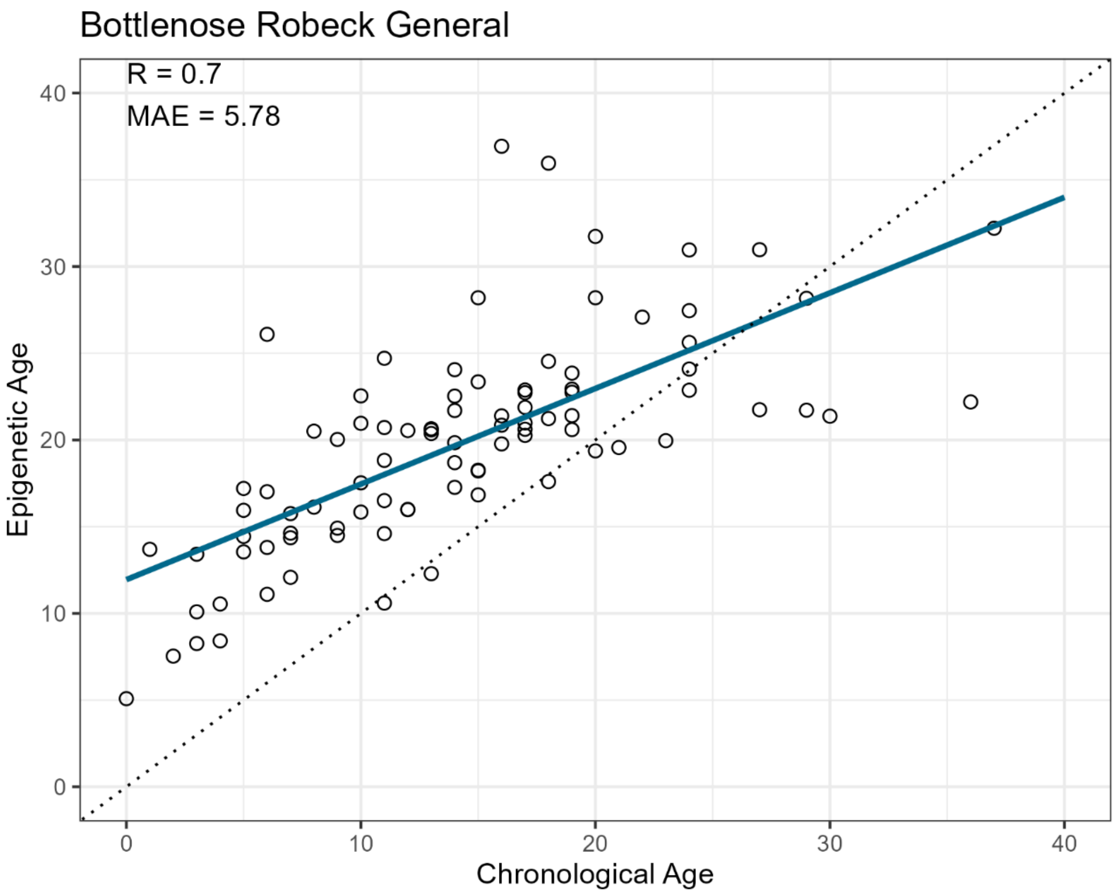

Supplementary Fig. 8. Epigenetic age versus chronological age for Shark Bay Indo-Pacific bottlenose dolphins (*Tursiops aduncus*) using Robeck et al.'s (2021) bottlenose dolphin general clock. Epigenetic ages calculated for 68 individuals totaling 90 samples which were included in our calibration dataset, including precision (R) and accuracy in years (MAE) for this epigenetic clock. The regression line is shown in blue. The dotted diagonal line indicates epigenetic age = chronological age.

Supplementary Table 10. Results of LMM using epigenetic age estimates from Robeck et al.'s (2021) bottlenose dolphin general clock [3].

**Predictor variable**	**b**	***p*-value**
Epigenetic age *($N_{Samples}$= 50, N_{Ind}= 38)*		
Intercept	10.12	<0.0001*
Cumulative social bond strength	-12.46	0.08
Mean male group size	0.53	0.40
Chronological age	0.82	<0.0001*

*Significant at $p < 0.05$

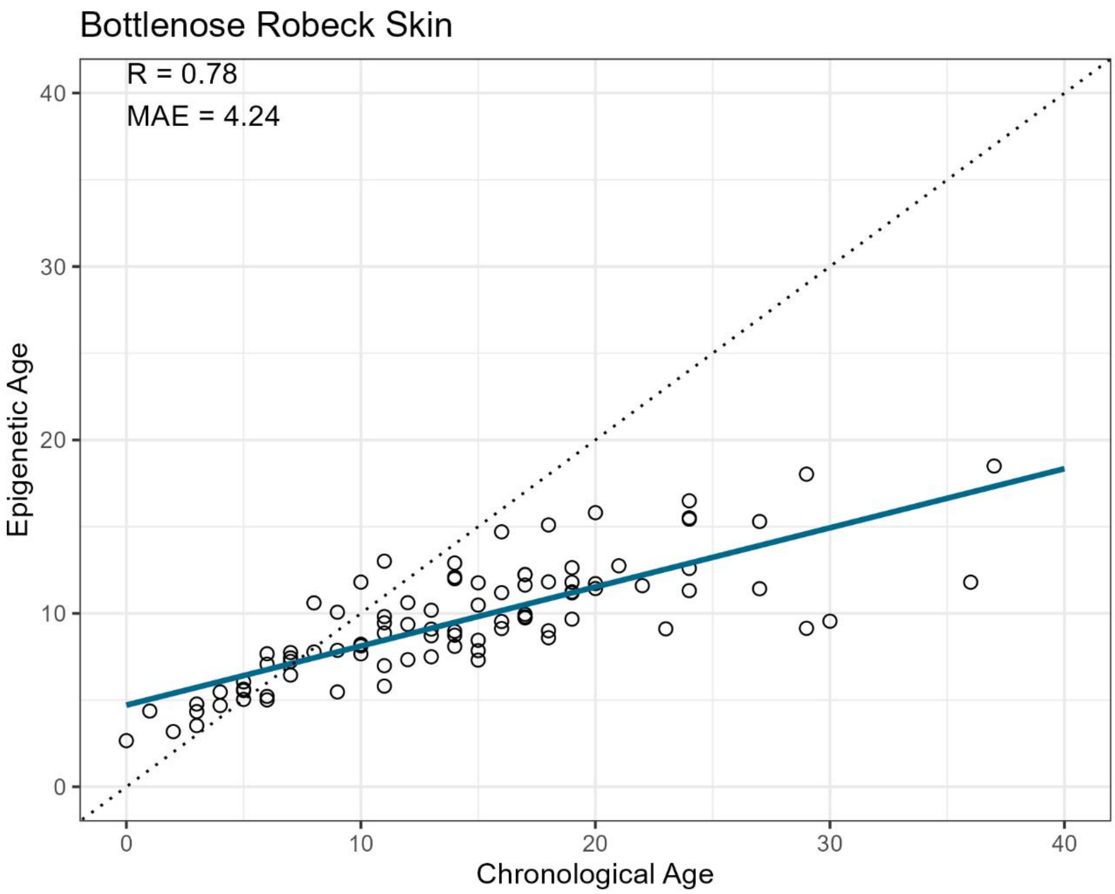

Supplementary Fig. 9. Epigenetic age versus chronological age for Shark Bay Indo-Pacific bottlenose dolphins (*Tursiops aduncus*) using Robeck et al.'s (2021) bottlenose dolphin skin clock. Epigenetic ages calculated for 68 individuals totaling 90 samples which were included in our calibration dataset, including precision (R) and accuracy in years (MAE) for this epigenetic clock. The regression line is shown in blue. The dotted diagonal line indicates epigenetic age = chronological age.

Supplementary Table 11. Results of LMM using epigenetic age estimates from Robeck et al.'s (2021) bottlenose dolphin skin clock [3].

**Predictor variable**	**b**	***p*-value**
Epigenetic age *($N_{Samples}$= 50, N_{Ind}= 38)*		
Intercept	4.58	<0.0001*
Cumulative social bond strength	-3.17	0.29
Mean male group size	0.22	0.41
Chronological age	0.37	<0.0001*

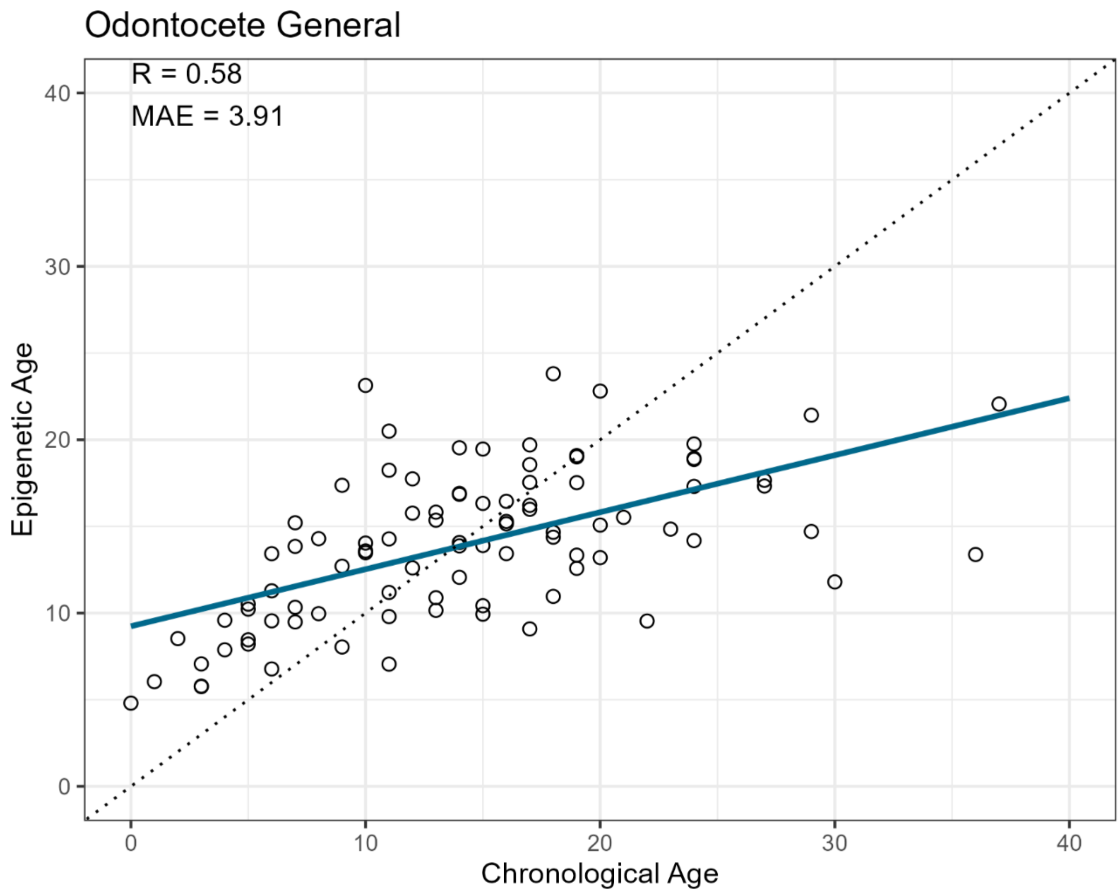

Supplementary Fig. 10. Epigenetic age versus chronological age for Shark Bay Indo-Pacific bottlenose dolphins (*Tursiops aduncus*) using Robeck et al.'s (2021) odontocete general clock. Epigenetic ages calculated for 68 individuals totaling 90 samples which were included in our calibration dataset, including precision ® and accuracy in years (MAE) for this epigenetic clock. The regression line is shown in blue. The dotted diagonal line indicates epigenetic age = chronological age.

Supplementary Table 12. Results of LMM using epigenetic age estimates from Robeck et al.'s (2021) odontocete general clock [4].

**Predictor variable**	**b_{median}**	**2.5% – 97.5%**
Epigenetic age *($N_{Samples}$= 50, N_{Ind}= 38)*		
Intercept	10.41	<0.0001*
Cumulative social bond strength	2.16	0.67
Mean male group size	0.10	0.82
Chronological age	0.23	0.051

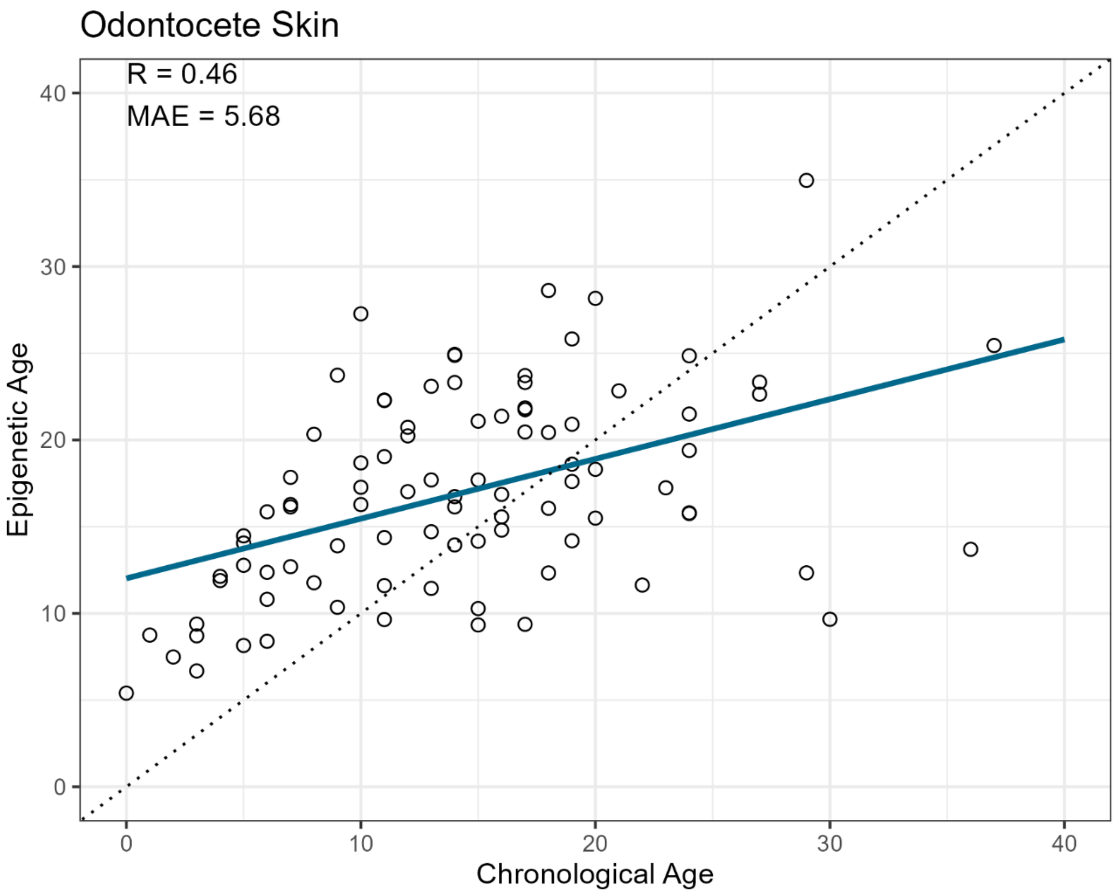

Supplementary Fig. 11. Epigenetic age versus chronological age for Shark Bay Indo-Pacific bottlenose dolphins (*Tursiops aduncus*) using Robeck et al.'s (2021) odontocete skin clock. Epigenetic ages calculated for 68 individuals totaling 90 samples which were included in our calibration dataset, including precision (R) and accuracy in years (MAE) for this epigenetic clock. The regression line is shown in blue. The dotted diagonal line indicates epigenetic age = chronological age.

Supplementary Table 13. Results of LMM using epigenetic age estimates from Robeck et al.'s (2021) odontocete skin clock [4].

**Predictor variable**	**b**	***p*-value**
Epigenetic age *($N_{Samples}$= 50, N_{Ind}= 38)*		
Intercept	12.13	<0.0001*
Cumulative social bond strength	7.51	0.30
Mean male group size	0.48	0.45
Chronological age	0.08	<0.0001*

*Significant at $p < 0.05$

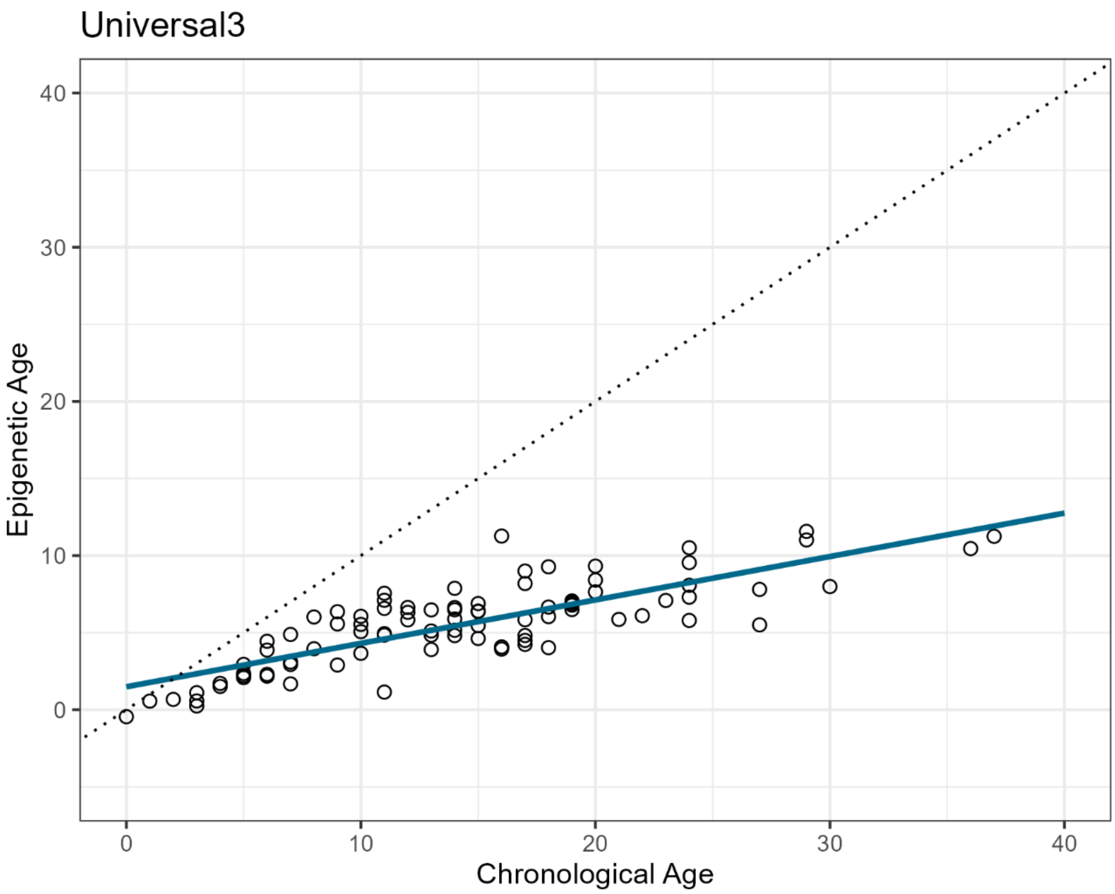

Supplementary Fig. 12. Epigenetic age versus chronological age for Shark Bay Indo-Pacific bottlenose dolphins (*Tursiops aduncus*) using Lu et al.'s (2023) universal clock 3. Epigenetic ages calculated for 68 individuals totaling 90 samples which were included in our calibration dataset, including precision (R) and accuracy in years (MAE) for this epigenetic clock. The regression line is shown in blue. The dotted diagonal line indicates epigenetic age = chronological age.

Supplementary Table 14. Results of LMM using epigenetic age estimates from Lu et al.'s (2023) universal 3 clock [5].

**Predictor variable**	**b**	***p*-value**
Epigenetic age *($N_{Samples}$= 50, N_{Ind}= 38)*		
Intercept	1.19	0.14
Cumulative social bond strength	-2.74	0.23
Mean male group size	0.21	0.29
Chronological age	0.29	<0.0001*

*Significant at $p < 0.05$

Data

Supplementary Data 1 (separate file)

Dataset including chronological and epigenetic age information, sex, and social variable estimates on all dolphins included in this study.

Supplementary Data 2 (separate file)

Dataset containing the SeSAMe [6] normalized beta values of all 90 samples of the 29,813 CpG sites mapping to the *T. aduncus* reference genome that were used for epigenetic clock calibration.

References

1 Krieger, N. *et al.* Use of Correct and Incorrect Methods of Accounting for Age in Studies of Epigenetic Accelerated Aging: Implications and Recommendations for Best Practices. *American Journal of Epidemiology* **192**, 800-811 (2023). https://doi.org/10.1093/aje/kwad025

2 Barratclough, A. *et al.* Accurate Epigenetic Aging in Bottlenose Dolphins (*Tursiops truncatus*), an Essential Step in the Conservation of at-Risk Dolphins. *Journal of Zoological and Botanical Gardens* **2**, 416-420 (2021).

3 Robeck, T. R. *et al.* Multi-tissue methylation clocks for age and sex estimation in the common bottlenose dolphin. *Frontiers in Marine Science* **8**, 713373 (2021).

4 Robeck, T. R. *et al.* Multi-species and multi-tissue methylation clocks for age estimation in toothed whales and dolphins. *Communications Biology* **4**, 642 (2021). https://doi.org/10.1038/s42003-021-02179-x

5 Lu, A. T. *et al.* Universal DNA methylation age across mammalian tissues. *Nature Aging* **3**, 1144-1166 (2023). https://doi.org/10.1038/s43587-023-00462-6

6 Zhou, W., Triche, T. J., Jr, Laird, P. W. & Shen, H. SeSAMe: reducing artifactual detection of DNA methylation by Infinium BeadChips in genomic deletions. *Nucleic Acids Research* **46**, e123-e123 (2018). https://doi.org/10.1093/nar/gky691
