# Supplementary material for: Social bonds decrease epigenetic age in male bottlenose dolphins
Source: Commun Biol. 2025 Nov 29;8:1765. doi: 10.1038/s42003-025-09227-w (PMC12700864; doi:10.1038/s42003-025-09227-w)
Supplement: Supplementary file 3 — Description of Additional Supplementary files [file 42003_2025_9227_MOESM3_ESM.pdf]

Description of Additional Supplementary files

File name: Supplementary Data 1

Description: Dataset including chronological and epigenetic age information, sex, and social variable estimates on all dolphins included in this study.

File name: Supplementary Data 2

Description: Dataset containing the SeSAMe 6 normalized beta values of all 90 samples of the 29,813 CpG sites mapping to the T. aduncus reference genome that were used for epigenetic clock calibration.
