# Supplementary material for: Social bonds decrease epigenetic age in male bottlenose dolphins
Source: Commun Biol. 2025 Nov 29;8:1765. doi: 10.1038/s42003-025-09227-w (PMC12700864; doi:10.1038/s42003-025-09227-w)
Supplement: Supplementary file 6 — Reporting Summary [file 42003_2025_9227_MOESM6_ESM.pdf]

nature portfolio

Corresponding author(s): Livia Gerber

Last updated by author(s): Nov 10, 2025

Reporting Summary

Nature Portfolio wishes to improve the reproducibility of the work that we publish. This form provides structure for consistency and transparency in reporting. For further information on Nature Portfolio policies, see our Editorial Policies and the Editorial Policy Checklist.

Statistics

For all statistical analyses, confirm that the following items are present in the figure legend, table legend, main text, or Methods section.

n/a	Confirmed	
☐	☒	The exact sample size (*n*) for each experimental group/condition, given as a discrete number and unit of measurement
☐	☒	A statement on whether measurements were taken from distinct samples or whether the same sample was measured repeatedly
☐	☒	The statistical test(s) used AND whether they are one- or two-sided<br>*Only common tests should be described solely by name; describe more complex techniques in the Methods section.*
☐	☒	A description of all covariates tested
☐	☒	A description of any assumptions or corrections, such as tests of normality and adjustment for multiple comparisons
☐	☒	A full description of the statistical parameters including central tendency (e.g. means) or other basic estimates (e.g. regression coefficient) AND variation (e.g. standard deviation) or associated estimates of uncertainty (e.g. confidence intervals)
☐	☒	For null hypothesis testing, the test statistic (e.g. F, t, r) with confidence intervals, effect sizes, degrees of freedom and P value noted<br>*Give P values as exact values whenever suitable.*
☒	☐	For Bayesian analysis, information on the choice of priors and Markov chain Monte Carlo settings
☒	☐	For hierarchical and complex designs, identification of the appropriate level for tests and full reporting of outcomes
☐	☒	Estimates of effect sizes (e.g. Cohen's *d*, Pearson's *r*), indicating how they were calculated

Our web collection on statistics for biologists contains articles on many of the points above.

Software and code

Policy information about availability of computer code

Data collection	Our behavioural data was collected in the field using ethograms and photo-id. Thus, no software or code were used.
Data analysis	To obtain epigenetic age estimates for additional clocks, we used the 'MammalMethylClock' R package v1.0.0 Other R packages used in our analyses included 'ggpubr' v0.6.0, 'ggplot2' v3.5.1, 'glmmTMB' v1.1.10, gridExtra' v2.3, 'car' v3.1-3, 'lme4' v1.1-35.5, 'lmerTest' v3.1-3, and 'mediation' v4.5.0. All analyses were conducted in R v.4.4.0.

For manuscripts utilizing custom algorithms or software that are central to the research but not yet described in published literature, software must be made available to editors and reviewers. We strongly encourage code deposition in a community repository (e.g. GitHub). See the Nature Portfolio guidelines for submitting code & software for further information.

Data

Policy information about availability of data

All manuscripts must include a data availability statement. This statement should provide the following information, where applicable:

- Accession codes, unique identifiers, or web links for publicly available datasets
- A description of any restrictions on data availability
- For clinical datasets or third party data, please ensure that the statement adheres to our policy

The epigenetic data used for this project is part of the data release from the Mammalian Methylation Consortium (https://clockfoundation.org/MammalianMethylationConsortium) and is also posted on the Gene Expression Omnibus website (complete dataset: GSE223748). Code for epigenetic clock

calibration and the statistical analyses described in this study is publicly available on https://github.com/ligerber/epiclock. The mammalian methylation array is available from the non-profit Epigenetic Clock Development Foundation.

Research involving human participants, their data, or biological material

Policy information about studies with human participants or human data. See also policy information about sex, gender (identity/presentation), and sexual orientation and race, ethnicity and racism.

Reporting on sex and gender	n/a
Reporting on race, ethnicity, or other socially relevant groupings	n/a
Population characteristics	n/a
Recruitment	n/a
Ethics oversight	n/a

Note that full information on the approval of the study protocol must also be provided in the manuscript.

Field-specific reporting

Please select the one below that is the best fit for your research. If you are not sure, read the appropriate sections before making your selection.

☐ Life sciences ☐ Behavioural & social sciences ☒ Ecological, evolutionary & environmental sciences

For a reference copy of the document with all sections, see nature.com/documents/nr-reporting-summary-flat.pdf

Ecological, evolutionary & environmental sciences study design

All studies must disclose on these points even when the disclosure is negative.

Study description	Our study is based on long-term behavioural and genetic data collected on the population of Indo-Pacific bottlenose dolphins (Tursiops aduncus) off Monkey Mia in the eastern gulf of Shark Bay. In this study, we used behavioural and genetic information of animals where ages were known with an accuracy of one year or less and that were observed at least 15 times prior to sampling.
Research sample	A population of wild Indo-Pacific bottlenose dolphins (Tursops aduncus) observed for more than 4 decades.
Sampling strategy	Behavioural data were collected based on standardised behavioural observations in the form of 5-minute surveys of opportunistically encountered groups in this population since 1984. Genetic sampling was opportunistic and started in 1994.
Data collection	Data is collected via standardised behavioural observations using an ethogram and photo-identification. Data has been collected over more than 40 years by a range of people including volunteers, graduate and undergraduate students, and principal investigators. The following authors of this study were involved in fieldwork: LG, SLK, SJA, RCC, KGH, and MK.
Timing and spatial scale	We used data that was collected as part of the long-term study on Shark Bay dolphins. Data collection started in 1984 and is still ongoing.
Data exclusions	As in all our studies, we excluded resightings of the same group repeadetdly encountered within 2 hours. We also removed all surveys in which the predominant behavioural activity was foraging (defined based on inter-individual spacing, relative orientation, dive type, and direct observations of prey or feeding), because animals tend to loosely aggregate in large groups at the same foraging patch but are not necessarily associating.
Reproducibility	This is a non-experimental study on wild animals using all data available. Therefore, reproducibility is not applicable.
Randomization	This is a non-experimental study on wild animals using all data available. Therefore, randomisation is not applicable.
Blinding	This is a non-experimental study on wild animals using all data available. Therefore, blinding is not applicable.

Did the study involve field work? ☒ Yes ☐ No

Field work, collection and transport

Field conditions	Field work is mostly carried out in the Austral winter between May and October. Behavioural data is collected whenever the weather

Field conditions	permits and Beaufort scales are below 3.
Location	Our field site is based in the waters off Monkey Mia in the eastern gulf of Shark Bay, Western Australia.
Access & import/export	Our samples are imported in line with CITES (since in effect).
Disturbance	Animals in our study population are well habituated to the presence of boats and dolphins are always approached at low speeds.

Reporting for specific materials, systems and methods

We require information from authors about some types of materials, experimental systems and methods used in many studies. Here, indicate whether each material, system or method listed is relevant to your study. If you are not sure if a list item applies to your research, read the appropriate section before selecting a response.

Materials & experimental systems

n/a	Involved in the study	
☒	☐	Antibodies
☒	☐	Eukaryotic cell lines
☒	☐	Palaeontology and archaeology
☐	☒	Animals and other organisms
☒	☐	Clinical data
☒	☐	Dual use research of concern
☒	☐	Plants

Methods

n/a	Involved in the study	
☒	☐	ChIP-seq
☒	☐	Flow cytometry
☒	☐	MRI-based neuroimaging

Animals and other research organisms

Policy information about studies involving animals; ARRIVE guidelines recommended for reporting animal research, and Sex and Gender in Research

Laboratory animals	n/a
Wild animals	This study is based on long-term behavioural and genetic data collected on the population of Indo-Pacific bottlenose dolphins (Tursiops aduncus) off Monkey Mia in the eastern gulf of Shark Bay, Western Australia. Standardised behavioural observations in the form of 5-minute surveys of opportunistically encountered groups have been conducted on this population since 1984.
Reporting on sex	We included only males to investigate the relationship between social bonds and epigenetic age. This is because males, but not females, in our study population are characterised by a highly complex, multi-level social system.<br>To design the epigenetic clock, samples of both sexes were included.
Field-collected samples	We obtained skin biopsy samples from dolphins on an opportunistic basis using a purpose-designed system for sampling small cetaceans.
Ethics oversight	The study was carried out in compliance with the animal ethics policies of the University of Zurich, University of Bristol, and University of Western Australia.

Note that full information on the approval of the study protocol must also be provided in the manuscript.

Plants

Seed stocks	n/a
Novel plant genotypes	n/a
Authentication	n/a
